# Plasma metabolites distinguish dementia with Lewy bodies from Alzheimer’s disease: a cross-sectional metabolomic analysis

**DOI:** 10.3389/fnagi.2023.1326780

**Published:** 2024-01-04

**Authors:** Xiaobei Pan, Paul C. Donaghy, Gemma Roberts, Leonidas Chouliaras, John T. O’Brien, Alan J. Thomas, Amanda J. Heslegrave, Henrik Zetterberg, Bernadette McGuinness, Anthony P. Passmore, Brian D. Green, Joseph P. M. Kane

**Affiliations:** ^1^School of Biological Sciences, Queen’s University Belfast, Belfast, United Kingdom; ^2^Translational and Clinical Research Institute, Newcastle University, Newcastle upon Tyne, United Kingdom; ^3^Department of Psychiatry, University of Cambridge, Cambridge, United Kingdom; ^4^Department of Neurodegenerative Disease, University College London Queen Square Institute of Neurology, London, United Kingdom; ^5^Dementia Research Institute, UCL, London, United Kingdom; ^6^Department of Psychiatry and Neurochemistry, Institute of Neuroscience and Physiology, The Sahlgrenska Academy, University of Gothenburg, Gothenburg, Sweden; ^7^Clinical Neurochemistry Laboratory, Sahlgrenska University Hospital, Mölndal, Sweden; ^8^Hong Kong Center for Neurodegenerative Diseases, Kowloon, Hong Kong SAR, China; ^9^Wisconsin Alzheimer’s Disease Research Center, University of Wisconsin School of Medicine and Public Health, University of Wisconsin-Madison, Madison, WI, United States; ^10^Centre for Public Health, Queen’s University Belfast, Belfast, United Kingdom

**Keywords:** dementia, Lewy body dementia, Alzheimer’s disease, metabolomic analyses, biomarkers

## Abstract

**Background:**

In multifactorial diseases, alterations in the concentration of metabolites can identify novel pathological mechanisms at the intersection between genetic and environmental influences. This study aimed to profile the plasma metabolome of patients with dementia with Lewy bodies (DLB) and Alzheimer’s disease (AD), two neurodegenerative disorders for which our understanding of the pathophysiology is incomplete. In the clinical setting, DLB is often mistaken for AD, highlighting a need for accurate diagnostic biomarkers. We therefore also aimed to determine the overlapping and differentiating metabolite patterns associated with each and establish whether identification of these patterns could be leveraged as biomarkers to support clinical diagnosis.

**Methods:**

A panel of 630 metabolites (Biocrates MxP Quant 500) and a further 232 metabolism indicators (biologically informative sums and ratios calculated from measured metabolites, each indicative for a specific pathway or synthesis; MetaboINDICATOR) were analyzed in plasma from patients with probable DLB (*n* = 15; age 77.6 ± 8.2 years), probable AD (*n* = 15; 76.1 ± 6.4 years), and age-matched cognitively healthy controls (HC; *n* = 15; 75.2 ± 6.9 years). Metabolites were quantified using a reversed-phase ultra-performance liquid chromatography column and triple-quadrupole mass spectrometer in multiple reaction monitoring (MRM) mode, or by using flow injection analysis in MRM mode. Data underwent multivariate (PCA analysis), univariate and receiving operator characteristic (ROC) analysis. Metabolite data were also correlated (Spearman r) with the collected clinical neuroimaging and protein biomarker data.

**Results:**

The PCA plot separated DLB, AD and HC groups (R2 = 0.518, Q2 = 0.348). Significant alterations in 17 detected metabolite parameters were identified (*q* ≤ 0.05), including neurotransmitters, amino acids and glycerophospholipids. Glutamine (Glu; *q* = 0.045) concentrations and indicators of sphingomyelin hydroxylation (*q* = 0.039) distinguished AD and DLB, and these significantly correlated with semi-quantitative measurement of cardiac sympathetic denervation. The most promising biomarker differentiating AD from DLB was Glu:lysophosphatidylcholine (lysoPC a 24:0) ratio (AUC = 0.92; 95%CI 0.809–0.996; sensitivity = 0.90; specificity = 0.90).

**Discussion:**

Several plasma metabolomic aberrations are shared by both DLB and AD, but a rise in plasma glutamine was specific to DLB. When measured against plasma lysoPC a C24:0, glutamine could differentiate DLB from AD, and the reproducibility of this biomarker should be investigated in larger cohorts.

## Introduction

In clinical practice, dementia with Lewy bodies (DLB) is significantly under-detected. Neuropathology indicative of DLB is observed in over 25% of dementia cases (McAleese et al., 2021), yet DLB represents under 5% of cases diagnosed antemortem (Kane et al., 2018). Of these cases, more than half are initially mis-diagnosed as conditions other than DLB. Alzheimer’s disease (AD) is the most frequent initial misdiagnosis (Surendranathan et al., 2020). Accurately recognizing DLB remains a crucial objective because DLB is associated with shorter survival, increased hospitalization and greater carer stress than other types of dementia (Lee et al., 2013; Price et al., 2017; Mueller et al., 2018). DLB requires its own specific management approaches which further justify the need for better diagnosis (Taylor et al., 2020).

Our understanding of the multifactorial pathophysiology of DLB is incomplete. Although, like Parkinson’s disease (PD), DLB is primarily driven by the aggregation of α-synuclein into Lewy bodies and Lewy neurites, clinical expression is also significantly influenced by co-morbid AD pathology (Tiraboschi et al., 2006). The genetic architecture of DLB, while distinct from those of AD and PD, also overlaps with both disorders (Chia et al., 2021). A similar pattern is seen in the influence of non-genetic factors, where depression and low caffeine increase risk of DLB more strongly than they do in AD or PD. Smoking and education have opposing risk effects on PD and AD, but are not associated with a higher risk of DLB (Boot et al., 2013).

Although clinical features and aligned clinical tools can support routine detection of DLB, biomarkers play an important role in accurate diagnosis (McKeith et al., 2017; O’Brien et al., 2020; Surendranathan et al., 2021). Striatal and cardiac imaging modalities [^123^I-N-3-fluoropropyl-2β-carbomethoxy-3β-4-iodophenyl tropane single photon emission computed tomography (^123^I-FP-CIT SPECT)] and cardiac [^123^I-metaiodobenzylguanidine (MIBG) scintigraphy] offer good sensitivity and specificity (Kane et al., 2018), but are expensive and burdensome for patients, caregivers and services. Polysomnography is, like FP-CIT and MIBG, also included in the DLB diagnostic criteria, but access to all three varies regionally and internationally. In recent years blood-based biomarkers for neurodegenerative illness have emerged (Teunissen et al., 2022), however, plasma biomarkers for AD [the ratio of 42 to 40 amino acid-long amyloid β (Aβ42/40) and tau phosphorylated at amino acid 181 (p-tau181)], astrocyte-expressed proteins [glial fibrillary acidic protein (GFAP)] and neurodegeneration [neurofilament light (NfL)] have limited utility in differentiating DLB from AD (Chouliaras et al., 2022; Gonzalez et al., 2022; Hamilton et al., 2023).

Metabolomics is an emerging discipline dedicated to the study of small metabolites in cells, tissues and biofluids, using a comprehensive, simultaneous and systematic profiling of numerous metabolite concentrations (Menni et al., 2017). Minor changes to endogenous and environmental factors can be reflected downstream at the level of metabolites and metabolomics is therefore thought to possess the potential to create a convergence of genetic, environmental, and physiological elements to multifactorial diseases like DLB (Shao and Le, 2019). Furthermore, where aberrations are identified, they may be leveraged as biomarkers in diagnostic practice and precision medicine (Xiao et al., 2022).

Metabolomic-based biomarker research in PD has advanced considerably in recent years (Li et al., 2022). Metabolomic profiling of the plasma of patients with PD found dysregulation in kynurenine pathways when compared with healthy controls (Chang et al., 2018). This has led to the proposal that supplementation with kynurenic acid, or the reduction of quinolinic acid using kynurenine 3-monooxygenase inhibitors could be a viable therapeutic pathway for PD treatment (Shao and Le, 2019). A 2020 systematic review identified 11 studies investigating 22 metabolites in DLB cohort (Scholefield et al., 2020). Only one report analyzed plasma samples. This narrowly focused study measured just four nitric oxide metabolites in DLB plasma samples and compared them with healthy controls (Molina et al., 2002). No studies have adopted a metabolomic approach, nor investigated a role for metabolite biomarkers discriminating DLB from AD (Scholefield et al., 2020).

There remains an unexplored potential for metabolite alterations to improve our understanding of the pathology or to develop novel biomarkers of neurodegenerative diseases, particularly in DLB where there is a near complete lack of investigation. Herein, we employed a validated LC–MS/MS methodology to quantify the plasma levels of 630 annotated metabolites to compare the metabolome of DLB with that of both AD and healthy controls. A further 232 metabolism indicators were obtained by calculating established metabolite sums and ratios. The aim was to identify novel DLB biomarkers capable of distinguishing the disease from AD, and to corroborate these findings using known neuroimaging and protein biomarkers for DLB.

## Methods

### Recruitment

As part of two neuroimaging studies (Donaghy et al., 2018; Kane et al., 2019) subjects over 60 years old with probable AD (McKhann et al., 2011) or probable DLB (McKeith et al., 2017, p. 201) were recruited through psychiatry of old age, geriatric medicine, and neurology services in North-east England between 2013 and 2017. Cognitively healthy older adults who demonstrated no evidence of dementia or mild cognitive impairment, were recruited through local research registers or were relatives of participants with dementia. Ethical approval for the two studies contributing data to this project were awarded by an NHS Regional Ethics Committee (NRES Committee North East—Newcastle & North Tyneside; references 13/NE/0268 and 13/NE/0064).

After venipuncture and collection in EDTA tubes, samples were centrifuged to isolate plasma, aliquoted and stored at −70°C.

### Cognitive and clinical assessments

At baseline, a thorough assessment was carried out on each patient. Cognitive impairment was assessed in all groups with the Mini-mental State examination (Folstein et al., 1975) and revised Addenbrooke’s Cognitive Examination (Mioshi et al., 2006). Functional impairment in participants with AD and DLB was assessed using the Bristol and Instrumental Activities of Daily Living Scales (Lawton and Brody, 1969; Bucks et al., 1996). Core DLB symptoms of visual hallucinations, motor parkinsonism, REM sleep behavior disorder, and fluctuations in cognition and arousal were assessed in subjects with DLB and AD using the hallucinations subscale of the neuropsychiatric inventory (NPI; Cummings, 1997), the Unified Parkinson’s Disease Rating Scale motor subscale (MDS-UPDRS) (Goetz et al., 2008), Mayo Sleep Questionnaire (Boeve et al., 2011), and Dementia Cognitive Fluctuations Scale (Lee et al., 2014) respectively.

### MIBG cardiac scintigraphy

Most subjects with probable AD (87%; 13/15) or probable DLB (93%; 14/15) underwent MIBG cardiac scintigraphy, which is an indicative biomarker of DLB. A reduction in the ratio of cardiac MIBG uptake to a mediastinal reference point [heart:mediastinum ratio (HMR)] below a predetermined threshold is considered representative of sympathetic denervation and suggestive of DLB. Details of image acquisition and HMR analysis has been previously published (Kane et al., 2019).

### Protein biomarker analysis

Protein biomarker measurements were conducted at the UK Dementia Research Institute biomarker laboratory as previously published (Chouliaras et al., 2022). In brief, commercially available Single molecule array (Simoa) assays were used to measure plasma Aβ40, Aβ42, Aβ40/42, GFAP, NfL and p-tau181 concentrations on an HDx instrument (Quanterix, Billerica, MA).

### Targeted metabolomics analysis

Targeted metabolomics profiling was performed using a commercially available MxP Quant500 kit (Biocrates Life Science AG, Innsbruck, Austria), which quantifies 630 metabolites and lipids from 26 analyte classes. This process also permits the calculation of 232 “metabolism indicators.” These are pre-determined sums and ratios comprising the quantified metabolites and pertain to specific biological pathways or syntheses.

All frozen plasma samples (−80°C) were thawed on ice before preparation, according to the instruction from the kit manufacturer. In brief, 10 μL of phosphate-buffered saline, calibrators, quality controls (QCs, lyophilized plasma spiking with metabolites at three known concentrations), and plasma samples were added to a 96-well plate which contains isotopic-labeled internal standards, followed by adding phenyl isothiocyanate (PITC) to derivatize amino acids and biogenic amines. Metabolites separation was performed using an ultra-performance liquid chromatography (UPLC) system (AB SCIEX ExionLC system, California, United States) with a reversed-phase MxP Quant 500 UHPLC column and analyzed using a triple-quadrupole mass spectrometer (Xevo TQ-S, Waters Corporation, Milford, United States) operating in the multiple reaction monitoring (MRM) mode. All the other metabolites (acylcarnitines, hexoses, glycerophospholipids, and sphingolipids) were quantified using the same mass spectrometer without column separation by the flow injection analysis (FIA) operating in MRM mode.

For quantitation, both LC and FIA data were converted and imported directly into the Biocrates software, MetIDQ Oxygen, and quantified. MetIDQ includes an automated simple target normalization procedure based on QC or sample pool for batch-to-batch and kit plate-to-plate correction for sample cohort across several kit plates. Metabolite concentrations were calculated and expressed as micromole (μM).

Whenever ≥20% of measurements for a metabolite were lower than the limit of detection (LOD), the metabolite was described as “undetectable” and was excluded from the analysis. Any metabolism indicators involving undetectable metabolites were not calculated. The LOD of each metabolite was based on the Quant500 kit methodology in accordance with the manufacturer’s instructions.

### Statistical analysis

The normality of individual metabolite was tested with the Shapiro–Wilk test using SPSS (version 26). The normally distributed data was analyzed using one-way ANOVA and Student’s t-test and non-distributed data analyzed using the Kruskal-Wallis test and multivariate analysis (PCA analysis) was performed using SIMCA 17. A heatmap was generated, and potential biomarkers and paired biomarker ratios were identified using MetaboAnalyst 5.0 (Pang et al., 2021). The receiver operating characteristic (ROC) analysis and bar chart was performed using GraphPad Prism 9. Spearman’s rank correlation coefficient was performed using SPSS (version 26) to determine the correlations between each metabolite and HMR, p-tau181, Aβ40, Aβ42, Aβ40/42, GFAP and NfL in AD and DLB subjects.

## Results

A total of 45 participants were included in the study. Table 1 summarizes the group characteristics. The groups were well matched for age and sex, and the AD and DLB well matched for levels of both cognitive and functional measures. Measures of parkinsonism, fluctuations and hallucinations were as expected all higher in subjects with DLB than those with AD.

**Table 1:** Clinical and demographic characteristics of participants.

	AD	DLB	Cont	*p*
n	15	15	15	
Age at consent, mean (±SD)	76.1 (6.4)	76.4 (7.7)	75.2 (6.9)	0.89^a^, 0.92^b^
Sex, number male (%)	12 (80)	13 (87)	11 (73)	0.66^a^, 1.00^b^
ACE-R, mean (±SD)	66.8 (13.9)	67.2 (13.8)	94.8 (2.8)	**<0.01**^**a**^, 0.94^b^
MMSE, mean (±SD)	22.3 (3.3)	21.9 (5.2)	29.0 (1.1)	**<0.01**^**a**^, 0.80^b^
MDS-UPDRS, mean (±SD)	6.2 (6.9)	33.3 (24.6)	5.3 (3.1)	**<0.01**^**a**^,**<0.01**^**b**^
NPI hallucinations subscale, mean (±SD)	0.0 (0.0)	2.1 (2.5)	–	**<0.01**^**b**^
DCFS, mean (±SD)	6.8 (2.3)	11.2 (3.3)	–	**<0.01**^**b**^
MSQ Dream enactment (% affirmative)	2 (13)	4 (27)	–	0.65^b^
BADL, mean (±SD)	11.4 (7.8)	19.9 (11.8)	–	0.03^b^
IADL, mean (±SD)	5.7 (4.5)	4.4 (5.6)	–	0.52^b^

ACE-R, Addenbrooke’s cognitive examination – revised; AD, Probable Alzheimer’s disease; BADL, Bristol Activities of Daily Living Scale; Cont, Cognitively healthy controls; DCFS, Dementia Cognitive Fluctuations Scale; DLB, Probable dementia with Lewy bodies; IADL, Instrumental Activities of Daily Living Scale; MMSE, Minimental state examination; MSQ, Mayo Sleep Questionnaire; NPI, Neuropsychiatric inventory; SD, standard deviation; UPDRS, Unified Parkinson’s Disease Rating Scale motor subscale. ^a^AD/DLB/HC; ^b^AD/DLB. Bold values are statistically significant.

### Profiling of the plasma metabolome

**Figure 1:**
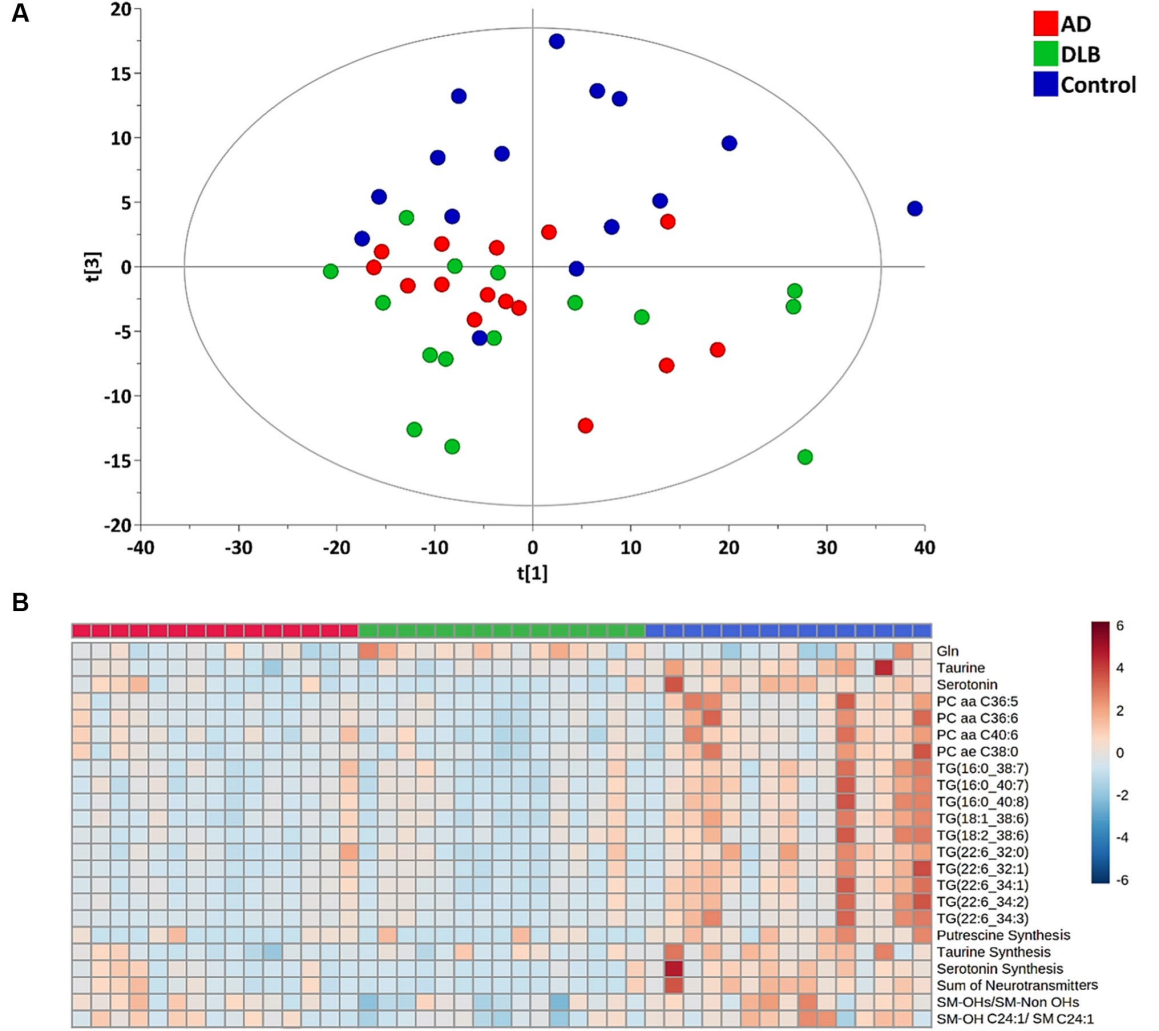
**(A)** PCA plot of plasma metabolites profiling for the healthy controls, AD and DLB groups. **(B)** Heatmap for significantly changed metabolites/metabolism indicators among healthy controls, AD and DLB groups with the *q* ≤ 0.05.

Of 630 targeted metabolites, 530 were detectable and 100 undetectable. From the multivariant analysis, the principal component analysis (PCA) plot (Figure 1A) showed separation between control and AD/DLB groups with the R2 = 0.518 and Q2 = 0.348.

Among 530 detected metabolites, 17 metabolites, including glutamine, taurine, serotonin, four phosphatidylcholines and 10 triglycerides (Table 2) showed significant difference among three groups with the false discovery rate (FDR) ≤ 0.05. The heatmap (Figure 1B) demonstrates that these four phosphatidylcholines and 10 triglycerides all significantly decreased in both subjects with AD and with DLB. Glutamine was the only amino acid significantly elevated in DLB, but not in AD or controls. Of the 232 metabolism indicators (i.e., metabolite sums and ratios) 197 were determinable. Of these metabolism indicators four (sum of neurotransmitters, serotonin synthesis, taurine synthesis and putrescine synthesis) were significantly reduced in AD and DLB patients compared with controls. The ratio of total hydroxylated sphingomyelin to total sphingomyelin (SM-OH/SM) and also SM-OH C24:1/SM C24:1 decreased significantly in DLB, but not AD groups.

**Table 2:** Significantly altered metabolites/metabolism indicators with FDR values ≤0.05.

Metabolites/metabolism indicators	Value of *p*	FDR (q)	Tukey’s HSD
TG(22:6_34:1)	1.26E-05	0.003	DLB-Ctrl; AD-Ctrl
TG(18:1_38:6)	1.96E-05	0.003	DLB-Ctrl; AD-Ctrl
Sum of neurotransmitters (Dopamine + histamine + serotonin)	3.27E-05	0.015(K-W)	DLB-Ctrl; AD-Ctrl
Serotonin	3.94E-05	0.015(K-W)	DLB-Ctrl; AD-Ctrl
TG(22:6_34:3)	7.50E-05	0.015(K-W)	DLB-Ctrl; AD-Ctrl
PC aa C36:5	7.61E-05	0.007	DLB-Ctrl; AD-Ctrl
Taurine	8.76E-05	0.015(K-W)	DLB-Ctrl; AD-Ctrl
Serotonin synthesis (Serotonin/tryptophan)	1.01E-04	0.015(K-W)	DLB-Ctrl; AD-Ctrl
TG(16:0_40:7)	1.53E-04	0.009	DLB-Ctrl; AD-Ctrl
PC aa C40:6	1.78E-04	0.010	DLB-Ctrl; AD-Ctrl
TG(22:6_32:1)	3.15E-04	0.034(K-W)	DLB-Ctrl; AD-Ctrl
TG(22:6_34:2)	3.47E-04	0.034(K-W)	DLB-Ctrl; AD-Ctrl
PC ae C38:0	4.93E-04	0.018	DLB-Ctrl; AD-Ctrl
PC aa C36:6	5.57E-04	0.041(K-W)	DLB-Ctrl; AD-Ctrl
TG(18:2_38:6)	7.42E-04	0.043(K-W)	DLB-Ctrl; AD-Ctrl
Taurine Synthesis (Taurine/cysteine)	7.55E-04	0.024	DLB-Ctrl; AD-Ctrl
TG(16:0_38:7)	9.41E-04	0.043(K-W)	DLB-Ctrl; AD-Ctrl
Putrescine Synthesis (Putrescine/ornithine)	9.67E-04	0.043(K-W)	DLB-Ctrl; AD-Ctrl
TG(16:0_40:8)	1.03E-03	0.043(K-W)	DLB-Ctrl; AD-Ctrl
TG(22:6_32:0)	1.06E-03	0.043(K-W)	DLB-Ctrl; AD-Ctrl
SM-OH C24:1/ SM C24:1	1.45E-03	0.0376	DLB-Ctrl
SM-OHs/SM-Non OHs (SM (OH) C24:1/SM C24:1	1.55E-03	0.039	DLB-Ctrl
Glutamine	1.91E-03	0.045	DLB-AD; DLB-Ctrl

### Evaluation of the performance of potential biomarkers

**Figure 2:**
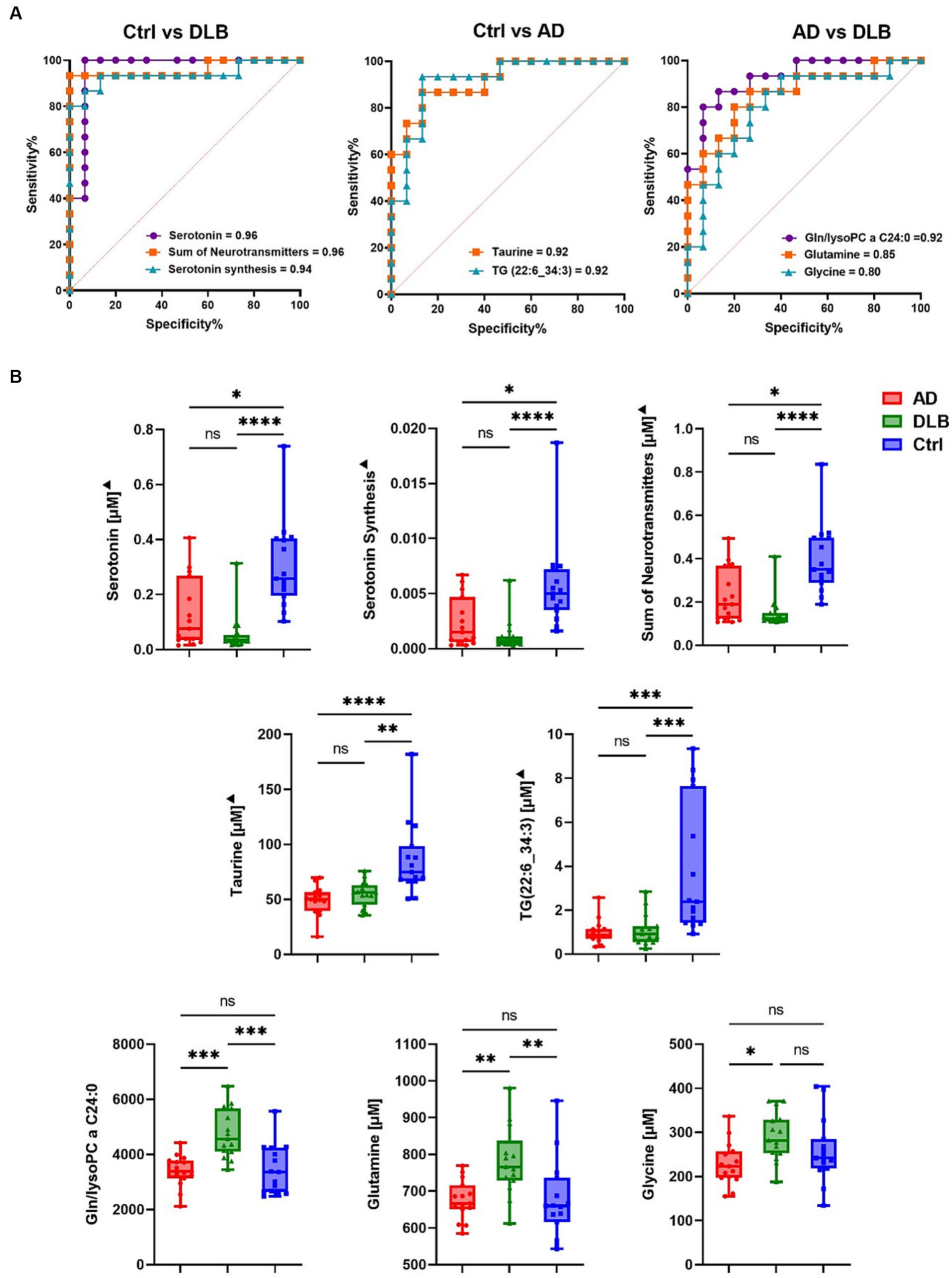
**(A)** ROC curve for potential biomarkers discriminating between every two groups. **(B)** Bar chart for potential biomarkers. The statistical analysis was performed using Krustal-Wallis test.

Receiver operating characteristic (ROC) analysis was performed to evaluate these metabolites as potential biomarkers. Serotonin and the sum of neurotransmitters (dopamine, histamine and serotonin) showed the highest discrimination ability between control and DLB groups with an AUC of 0.96 (sensitivity = 1.00, specificity = 0.90; Figure 2A). The serotonin synthesis indicator (serotonin/tryptophan) also discriminated these two groups with an AUC of 0.94 (sensitivity = 0.90, specificity = 0.90; Figure 2A). Two metabolites, taurine, and TG(22:6_34:3) could differentiate between control and AD groups with AUC of 0.92 (sensitivity = 0.90, specificity = 0.90; Figure 2A). No single metabolite or metabolism indicator was found to differentiate AD from DLB groups with AUC > 0.9 (Figure 2B). However, of the paired biomarker ratios generated by MetaboAnalyst, the ratio of glutamine to lysophosphatidylcholine C24:0 (lysoPC a 24:0) was the most optimal discriminator of DLB from AD. This achieved an AUC of 0.92 (sensitivity = 0.90 and specificity = 0.90; Figure 2A). Glutamine and glycine differentiated between DLB and AD with AUC of 0.85 (sensitivity = 0.80 and specificity = 0.80) and 0.80 (sensitivity = 0.70 and specificity = 0.80), respectively.

**Figure 3:**
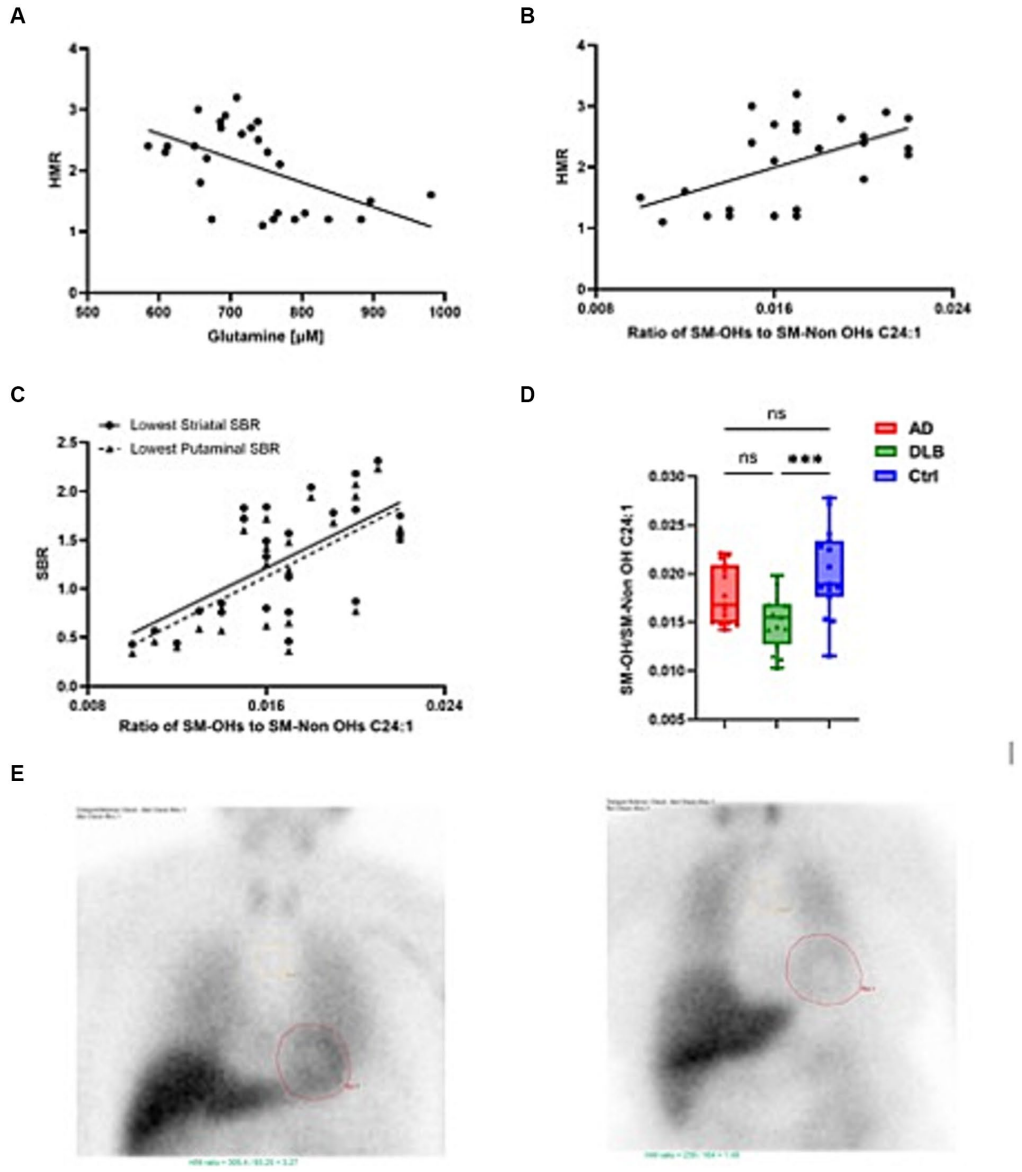
Plasma metabolites closely associated with heart:mediastinum ratio (HMR). **(A)** glutamine significantly negatively correlated with HMR **(B)** The ratio of SM-OHs/SM-Non OHs C24:1 significantly positively correlated with HMR **(C)** The ratio of SM-OHs/SM-Non OHs C24:1 significantly positively correlated with lowest SBR and **(D)** levels tended to be lower in cases of DLB. **(E)** shows MIBG scans of HMRs equivalent to 3.27 [normal, suggestive of AD in this study (left)] and 1.46 [abnormal, suggestive of DLB (left)].

The correlation between those significantly altered metabolites/metabolism indicators and HMR and plasma markers, including HMR, p-tau181, Aβ40, Aβ42, Aβ40/42, GFAP and NfL were investigated in exploratory analyses (Table 3). There was no significant correlation between p-tau181 and any of altered metabolites/metabolism indicators. Glutamine negatively associated with HMR (*p* = 0.004; *r* = −0.536) and positively associated with NfL (*p* = 0.002, *r* = 0.566) (Figure 3). Taurine and serotonin both negatively correlated with Aβ. Triglycerides negatively associated with NfL levels, while putrescine synthesis positively associated with NfL levels (*p* = 0.039, *r* = 0.392). Ratio of SM-OHs to SM-Non-OHs positively correlated with HMR (*p* = 0.015, *r* = 0.464) and negatively correlated with GFAP (*p* = 0.028, *r* = −0.415) and NfL (value of *p* = 0.009, *r* = −0.486), meanwhile the ratio of SM-OH C24:1/SM C24:1 similarly showed significant positive correlation to HMR (*p* = 0.005, *r* = 0.523) and negative correlation to GFAP (*p* = 0.018, *r* = −0.445) and NfL (*p* = 0.001, *r* = −0.591).

**Table 3:** Correlation analysis for the significantly altered metabolites/metabolism with HMR, Aβ40, Aβ42, Aβ42/Aβ40, p-tau181, GFAP and NfL.

		HMR	Aβ40	Aβ42	Aβ42/ Aβ40	p-tau181	GFAP	NfL	Striatal R SBR	Striatal L SBR	Putaminal R SBR	Putaminal L SBR
Glutamine	Pearson r	−0.536**	−0.186	−0.173	0.011	0.126	0.319	0.566**	−0.588**	−0.521**	−0.593**	−0.510**
	value of p	0.004	0.342	0.378	0.962	0.524	0.098	0.002	**0.002**	**0.008**	**0.002**	**0.009**
Taurine	Pearson r	−0.235	−0.448*	−0.421*	0.026	−0.148	−0.274	0.032	−0.205	−0.296	−0.224	−0.302
	value of p	0.239	0.017	0.026	0.894	0.452	0.158	0.873	0.326	0.151	0.282	0.142
Serotonin	Pearson r	0.279	−0.401*	−0.532**	−0.273	0.012	−0.06	−0.191	0.410	0.359	0.407*	0.337
	value of p	0.160	0.034	0.004	0.160	0.951	0.761	0.331	0.042	0.078	0.043	0.100
PC ae C38:0	Pearson r	0.090	−0.306	−0.391*	−0.192	0.130	−0.013	−0.192	0.142	0.161	0.157	0.158
	value of p	0.657	0.113	0.040	0.327	0.509	0.947	0.327	0.498	0.441	0.723	0.454
TG(16:0_40:8)	Pearson r	0.094	−0.163	−0.245	−0.140	−0.346	−0.303	−0.397*	−0.053	−0.063	−0.050	−0.075
	value of p	0.642	0.406	0.209	0.479	0.071	0.117	0.037	0.801	0.765	0.812	0.720
TG(18:1_38:7)	Pearson r	0.192	−0.139	−0.143	−0.010	−0.223	−0.304	−0.389*	−0.038	0.044	−0.001	0.068
	value of p	0.338	0.480	0.469	0.961	0.255	0.116	0.041	0.858	0.835	0.995	0.746
TG(18:3_38:6)	Pearson r	0.357	−0.213	−0.358	−0.286	0.018	0.035	−0.399*	0.328	0.354	0.341	0.338
	value of p	0.068	0.276	0.062	0.141	0.928	0.859	0.035	0.109	0.083	0.095	0.099
Putrescine synthesis	Pearson r	−0.266	0.166	0.223	0.113	−0.054	−0.165	0.392*	−0.208	0.202	−0.234	−0.237
	value of p	0.181	0.400	0.254	0.567	0.786	0.402	0.039	0.318	0.333	0.260	0.254
Taurine synthesis	Pearson r	−0.111	−0.488**	−0.365	0.233	−0.194	−0.323	−0.048	−0.230	−0.311	−0.245	−0.315
	value of p	0.580	0.008	0.056	0.232	0.323	0.093	0.809	0.368	0.130	0.238	0.125
Serotonin synthesis	Pearson r	0.249	−0.428*	−0.539**	−0.275	0.087	−0.012	−0.084	0.357	0.327	0.355	0.309
	value of p	0.210	0.023	0.003	0.156	0.660	0.95	0.67	0.080	0.111	0.082	0.132
Sum of neurotransmitters	Pearson r	0.285	−0.392*	−0.526**	−0.275	0.075	−0.049	−0.158	0.429*	0.373	0.425*	0.349
	value of p	0.149	0.039	0.004	0.156	0.706	0.806	0.421	0.032	0.066	0.034	0.087
SM-OHs/SM-Non OHs	Pearson r	0.464*	0.018	0.08	0.091	0.177	−0.415*	−0.486**	0.491*	0.525*	0.509**	0.521**
	value of p	0.015	0.926	0.687	0.644	0.368	0.028	0.009	0.013	0.007	0.009	0.008
SM-OH/SM-Non OH C24:1	Pearson r	0.523**	−0.169	−0.099	0.12	0.067	−0.445*	−0.591**	0.626*	0.620*	0.645**	0.628**
	value of p	0.005	0.391	0.617	0.543	0.736	0.018	0.001	0.001	0.001	0.001	0.001

Bold values are statistically significant.

## Discussion

In this study we profiled the plasma metabolome of patients with DLB and AD, as well as cognitively normal control subjects, and determined the overlapping and differentiating metabolite patterns associated with these neurodegenerative diseases. Secondly, we determined whether identification of these patterns could be leveraged as biomarkers to support clinical diagnosis.

We observed 21 metabolites that were altered in both DLB and AD compared with healthy controls. Many of these perturbations relate to a reduction in circulating neurotransmitters, particularly serotonin.

These would seemingly arise from a decreased biosynthesis, but might also arise from other factors related to DLB and AD, such as medication or dietary intake. Taurine levels were reduced in both DLB and AD, but there were no differences between DLB and AD groups with respect to ‘taurine synthesis’ as a metabolic indicator. The likelihood is that these changes represent non-disease specific metabolic perturbations resulting from neurodegenerative processes, particularly since several of them moderately correlate with NfL, which is a recognized marker of neuroaxonal injury (Gaetani et al., 2019). The list of changes included putrescine synthesis, triglyceride homeostasis [TG (16:0_40:8), TG(18:1_38:7), TG(18:3_38:6)], and for sphingomyelin hydroxylation [SM-OH/SM-Non OH 24:1 and SM-OHs/SM-Non OHs]. Interestingly, these significantly correlated with GFAP, which is proposed to reflect neuroinflammation in the early stages of neurodegenerative disease (Surendranathan et al., 2018; Hansson, 2021; Loveland et al., 2023).

Given that the majority of cases with DLB demonstrate concomitant amyloid pathology it is unsurprising AD and DLB groups possessed similar metabolomic signatures (McAleese et al., 2021). We noted tendencies for metabolite concentrations to decrease in DLB and to be more pronounced than in AD, however, none of these reached statistical significance. Glutamine, which was significantly higher in DLB cases, was the only amino acid to significantly differ between AD and DLB. This alteration appears quite specific, given that glutamine correlated negatively with HMR (the semi-quantitative proxy measure of cardiac denervation derived from cardiac scintigraphy). Additionally, glutamine also correlated with both striatal left and right SBR and also putaminal left and right SBR (semi-quantitative measures of FP-CIT uptake).

Glutamine is an abundant free amino acid, which has a diverse set of functions in neuronal homeostasis that include its role as a fundamental precursor of neurotransmitters γ-aminobutyric acid (GABA) and glutamate. Alterations in plasma and CSF glutamine have been previously observed in numerous neurodegenerative diseases (Chang et al., 2018; Van Der Lee et al., 2018; Klatt et al., 2021), and it is therefore not unexpected therefore that glutamine correlated with NfL. What is less clear is why glutamine was significantly higher in DLB that in AD, particularly as both higher and lower levels have been observed in patients with PD (Klatt et al., 2021) and that the glutamine correlated with both semi-quantitative measures of indicative DLB biomarkers. One possible explanation may be a compensatory upregulation in glutamine synthesis in response to disruption of its physiological role in inhibiting α-synuclein aggregation (Wang et al., 2017).

Glutamine is also believed be neuroprotective against amyloid aggregation (Chen and Herrup, 2012). Although not noted in this study to correlate with protein markers of amyloid, AD requires the presence of amyloid pathology, while DLB does not (Jack et al., 2016). Another possibility is that elevated glutamine may reflect symptoms (such as parkinsonism or depressive illness) or their treatments (such as levodopa or antidepressants) recognized as more common in DLB than in AD. A larger sample size may have permitted detection of associated particular abnormalities in glutamine metabolism, such as glutaminase and glutamine synthetase activity.

None of the metabolic alterations identified in AD subjects (*n* = 40) enrolled to a recent Japanese study – ornithine, uracil, and lysine - were replicated in this study. The same study also did not find any association between AD and glutamine (Ozaki et al., 2022). We did not identify significant correlations between any metabolite or metabolomic marker and p-tau181, which has been observed at lower plasma concentrations in subjects with DLB than those with AD among 987 participants from the European DLB consortium (Gonzalez et al., 2022). This again may reflect the small sample size used in this study but also reinforces Gonzalez and colleagues’ assertion that plasma p-tau181 has limited diagnostic utility in differentiating DLB from AD (Chouliaras et al., 2022; Gonzalez et al., 2022).

A particularly intriguing finding of the present study was that the ratio of hydroxylated sphingomyelins to non-hydroxylated sphingomyelins (9-OH C24:1/SM C24:1) was significantly decreased in DLB subjects, but not AD subjects, compared with controls. This metabolic indicator correlated positively with both HMR and SBR, both specifically decreased in DLB. Sphingomyelin is a dominant sphingolipid of the major constituents of cell membranes (Gault et al., 2010). It is particularly enriched in the central nervous system as sphingomyelins, especially in hydroxylated form, are pivotal components of the myelin sheath that surrounds neuronal axons. Decreases in plasma sphingomyelins, and increases in their metabolite ceramide, have been consistently observed in AD, and direct and indirect links with amyloid and tau pathology described (Mielke and Haughey, 2012). Increases in serum sphingomyelin and plasma ceramide have also been investigated in PD (Alimov et al., 1990; Mielke et al., 2013), and glucocerebrosidase (GBA) mutations, a genetic risk factor for PD, implicated in ceramide metabolism (Sidransky et al., 2009). Of several enzymes involved in sphingomyelin metabolism, disruption of acid sphingomyelinase (ASMase) activity is implicated in accumulation of α-synuclein and mutations in the gene coding for ASMase is associated with earlier onset of PD (Alcalay et al., 2019; Usenko et al., 2022).

However, to our knowledge the hydroxylation status of sphingomyelin has not been specifically investigated in DLB, AD or PD. Fatty acid-2 hydroxylase (FA2N) is the enzyme responsible for synthesis of hydroxylated sphingomyelins, which are low-abundant but common sphingomyelins (Hama, 2010; Sessa et al., 2021) considered essential in maintaining stability of the myelin sheath (Signorelli et al., 2021). Mice deficient in FA2N have demonstrated age-related axon and myelin sheet degeneration that mirrors the negative correlation we observed between SM-OH C24:1/SM C24:1 and NfL (Zöller et al., 2008). Sphingomyelin hydroxylation should be explored as a possible pathogenetic mechanism in PD and DLB in metabolomic studies involving these populations.

Our finding that a combination of glutamine and lysoPC a C24:0 distinguished DLB from AD with 92% accuracy supports the concept that metabolomic analysis could be translated to the clinic. Here, it is glutamine which is the highly discriminative metabolite for these diseases. The plasma levels of lysoPC a C24:0 effectively normalized glutamine levels, and ultimately enhance its performance as a biomarker. LysoPC a C24:0 did not significantly differ between groups, although it tended to be lower in DLB. Therefore, in this context the lysoPC a C24:0 lipid species is not necessarily important in the pathology of either AD or DLB. That being said, lysoPC a C24:0 is a proinflammatory lipid and is believed to represent a marker of pericyte loss, vascular barrier disruption, and demyelination in neurodegenerative disease (Law et al., 2019). Increases in plasma lysoPC a C24:0 have been observed in AD samples when compared with age-matched controls, but more modest increases plasma concentrations also occurring with age (Dorninger et al., 2018).

Over half of DLB cases are initially misdiagnosed and is AD the disorder for which it is most frequently mistaken (Surendranathan et al., 2020). Although caveated by our small sample size, the 92% accuracy of a glutamine to lysoPC a C24:0 ratio observed in this study compares favorably with that of other DLB biomarkers. Three “indicative” DLB biomarkers; FP-CIT SPECT, cardiac MIBG, and polysomnography (PSG), are included in diagnostic criteria on the basis of utility in differentiating DLB and AD (McKeith et al., 2017). In an autopsy validated cohort, FP-CIT, a measurement of striatal dopaminergic activity, has demonstrated a sensitivity of 86% and a specificity of 83% (Walker and Walker, 2009). The only neuropathologically validated study of MIBG utility reports an accuracy of up to 94% (Matsubara et al., 2022), however, its specificity is believed to be lower in Western populations with higher rates of diabetes, cardiac disease and prescription of medications which might interfere with MIBG uptake. MIBG accuracy was 86% among a UK-based cohort of patients with DLB and AD (Kane et al., 2019). Irrespective of utility, all three indicative biomarkers are expensive, can be difficult for some patients to tolerate, and access to them varies considerably.

Blood-based biomarkers therefore offer a less invasive, clinically feasible alternative to imaging biomarkers. Although recent investigation of blood-based biomarkers has demonstrated differences between plasma concentration of phosphorylated tau in DLB and AD cohorts, neither demonstrated diagnostic accuracy for p-tau181 (AUC = 0.62–0.67) or p-tau231 (AUC = 0.56) supportive of their use as a biomarker (Chouliaras et al., 2022; Gonzalez et al., 2022). Our results demonstrate promise that plasma metabolomic analysis may feasibly translate into diagnostic use. It is important therefore that our results are replicated in larger cohorts, such as those curated by established DLB consortia (D’Antonio et al., 2021), and in datasets that include neuropathological validation of DLB diagnosis (Thomas et al., 2017).

### Strengths and limitations

To the best of our knowledge this study is the first to investigate the plasma metabolome of DLB and explore the potential of metabolites as novel disease biomarkers. Using well matched and well characterized cohorts, we adopted an established and replicable methodology with considerable scalability, using a more extensive panel of quantified metabolites than utilized in studies investigating PD or AD (Chang et al., 2022; Ozaki et al., 2022). The inclusion of wide range of calculated metabolite sums and ratios added further rigor to this investigation.

The main limitations of this pilot study are its small sample size and the absence of replication. Sample size may have precluded detection of metabolomic aberrations that might better explain our observations of disruptions in glutamine synthesis and sphingomyelin hydroxylation. Independent replication would have provided more certainty to our findings, particularly with respect to the accuracy of the glutamine to lysoPC a C24:0 ratio and identified false positives contributing to 92% accuracy observed in this sample. Subjects were categorized on the basis of clinical diagnosis rather than the gold standard of neuropathological examination; however, our clinical method has been validated against autopsy data (McKeith et al., 2000). Despite our detailed characterization of subjects, collection of CSF data and those from other DLB biomarkers (such as electroencephalogram and polysomnography), would likely have enhanced the accuracy of our diagnostic process. Baseline assessment was the only point at which samples were retrieved and were therefore unable to determine how metabolite profiles alter over the course of the disease. In this explorative study we did not adjust for age and sex, which may confound our findings, but groups were well-matched. The inclusion of a PD cohort, with or without dementia, would have helped provide additional insight into our results. Comparison of the PD metabolome with that of DLB may have helped determine whether the aberrations identified were driven by these two disorders’ overlapping neuropathology or by other factors, such as sequelae or treatment of dementia. Another limitation is that nutritional assessments were not conducted in this study, although it has been demonstrated malnutrition can be more common in DLB patients than in AD (Koyama et al., 2016). It also should be pointed out that the alteration of glutamine, glycine and taurine levels in AD patients’ plasma are independent of patients’ nutritional state (Aquilani et al., 2020).

Finally, selection bias is a potential factor in the interpretation of these findings; our DLB cohort was typical of those investigated throughout DLB literature, and we recognize that individuals with clinically silent DLB, particularly where AD pathology co-exists (Tiraboschi et al., 2006) will not be identified for participation in studies such as ours.

## Conclusion

This study indicates plasma metabolites correlate with imaging biomarkers of DLB, and this could be exploited as diagnostic biomarkers which differentiate DLB from AD. We assessed concentrations of 630 metabolites and 232 metabolism indicators, but a panel of five to 10 metabolites and metabolism indicators, if validated in a larger cohort, could provide scalable and cost-effective biomarkers which can be translated to the clinic.

## Data availability statement

The raw data supporting the conclusions of this article will be made available by the authors, without undue reservation.

## Ethics statement

The studies involving humans were approved by NHS Regional Ethics Committees (NRES Committee North East—Newcastle & North Tyneside; references 13/NE/0268 and 13/NE/0064). The studies were conducted in accordance with the local legislation and institutional requirements. The participants provided their written informed consent to participate in this study.

## Author contributions

XP: Formal analysis, writing – original draft, Visualization. PD: Writing – review & editing, Investigation. GR: Formal analysis, Investigation, Writing – review & editing. LC: Formal analysis, Writing – review & editing. JO’B: Funding acquisition, Writing – review & editing. AT: Funding acquisition, Writing – review & editing. AH: Formal analysis, Writing – review & editing. HZ: Funding acquisition, Writing – review & editing. BM: Funding acquisition, Writing – review & editing. AP: Funding acquisition, Writing – review & editing. BG: Conceptualization, Funding acquisition, Methodology, Writing – review & editing. JK: Conceptualization, Formal analysis, Funding acquisition, Investigation, Methodology, Writing – original draft, Writing – review & editing.
